# Burden of chronic pain among adult pastoralists in Ethiopia: a cross-sectional household survey

**DOI:** 10.1097/j.pain.0000000000003282

**Published:** 2024-06-05

**Authors:** Eleonore Baum, Sied Abdi, Jan Hattendorf, Peter van Eeuwijk, Rea Tschopp, Birgit Vosseler, Jakob Zinsstag, Nicole Probst-Hensch

**Affiliations:** a Swiss Tropical and Public Health Institute, Allschwil, Switzerland; b University of Basel, Basel, Switzerland; c Institute of Applied Nursing Sciences IPW, OST—Eastern Switzerland University of Applied Sciences, St.Gallen, Switzerland; d School of Nursing and Midwifery, Jigjiga University, Jigjiga, Ethiopia; e Institute of Social Anthropology, University of Basel, Basel, Switzerland; f Armauer Hansen Research Institute, Addis Ababa, Ethiopia

**Keywords:** Chronic pain, Ethiopia, Africa South of the Sahara, Somali people, Epidemiology, Minority groups

## Abstract

Chronic pain is a major global health problem. Untreated pain causes particular suffering in marginalized communities. Most studies investigating chronic pain in sub-Saharan Africa stem from South Africa and Nigeria. Pastoralists are particularly underrepresented in pain research. The main objective of this study is to investigate the burden of chronic pain in adult pastoralists in the Somali Regional State of Ethiopia. We conducted a cross-sectional household survey among adult pastoralists (aged 18 years or older, N = 299) by face-to-face interviews. To randomly select households, we applied GPS-based household localization and recruitment. Chronic pain was self-reported by 17.0% (95% CI: 10.8-25.7) of male and 34.7% (95% CI: 28.4-41.5) of female adult pastoralists. The prevalence of chronic pain increased with age from 5.4% (95% CI: 0.8-30.1; 18-34 years) to 27.1% (95% CI: 15.1-43.7; 35-54 years) to 69.1% (95% CI: 53.7-81.1; 55 years and older). The body sites most commonly affected among those with chronic pain were knees (37.2%), followed by lower back (33.7%) and head (23.3%). The data for the first time provide insights into the burden of chronic pain among Somali pastoralists and reveal associated risk factors. The results support the planning of locally adapted health interventions for pastoralist-specific pain management considering the effects of chronic pain on pastoralists' daily lives.

## 1. Introduction

Pain can be defined as an “unpleasant sensory and emotional experience associated with, or resembling that associated with, actual or potential tissue damage.”^46(p.14)^ Pain lasting longer than 3 months is considered chronic^52^ and a major global health problem.^13^ Worldwide, lower-back pain, migraine, and other musculoskeletal conditions have been highlighted as leading causes of years lived with disability.^16,23,26^

The burden of chronic pain is well described in high-income countries, but gaps exist for low- and middle-income countries (LMICs). Yet, their chronic pain burden is expected to increase primarily as a result of demographic aging,^22^ rising incidence of cancer,^50^ and limited access to treatment for severe pain in light of the opioid crisis.^34^ Furthermore, the global increase in mental disorders is likely to exacerbate chronic pain^43^ because of their comorbidity. Global inequalities regarding access to pain relief, however, are expected to not only persist but to increase.^12^

Only few studies on chronic pain have been conducted in sub-Saharan Africa.^30,55,58^ These studies often focused on chronic pain prevalence and failed to report on extended pain characteristics or treatment itineraries. As a limitation, Kamerman et al.^30^ highlighted that they did not measure chronic pain interference or its impact. Findings from other sub-Saharan African studies are often not generalizable to the general population.^58^

Data are particularly sparse for marginalized communities within LMICs including sub-Saharan Africa.^29,42^ Their suffering from pain seems much more pronounced,^14,33^ in part related to higher prevalence of road injuries, interpersonal and political violence, unregulated and physically straining manual labor, obstetric complications, and lacking access to adequate pain treatment.^18,35^

Pastoralists represent a marginalized population within LMICs and sub-Saharan Africa. They experience everyday hardships and difficulties in accessing biomedical care.^19,62^ This makes them vulnerable to poorer health outcomes,^44^ particularly women.^3^ Limited access to health care is associated with the development of chronic pain.^13^ Moreover, the practice of female genital cutting (FGC) is still common in East Africa.^17^ Having undergone FGC can cause debilitating pain.^45^

Pastoralists remain underrepresented in pain research. Existing studies commonly focus on communicable diseases.^19,56^ Therefore, it is difficult to translate research findings into targeted local practice.^27,38^ Considering the importance of traditional medicine^56^ and lay health knowledge,^40^ it is crucial to understand pastoralists' therapeutic practices to improve access to appropriate care.^41^ Filling the data gap is relevant because pain control is a basic health-related human right.^36^ Data obtained can help inform local policy that might improve prevention or treatment of pain while avoiding expensive and unnecessary biomedical care and interfering with the population's own potentially successful therapeutic practices.

The objectives are:

(1) To investigate the prevalence of chronic pain among adult pastoralists in the Somali Regional State (SRS) of Ethiopia and associated socioeconomic factors;
(2) To describe characteristics of chronic pain in terms of body sites, pain intensity, pain frequency, and impact on daily life; and
(3) To determine therapeutic practices of pastoralists with chronic pain.

## 2. Methods

### 2.1. Overall study design and population

This study is embedded into a larger consecutive mixed-methods research project. The overarching aim is to improve understanding of how pastoralists perceive, are burdened by, and deal with chronic pain.^5^ The project follows a transdisciplinary approach engaging academic and nonacademic stakeholders in a transformational research process coproduced by systemic and practical knowledge.^8,51,61^ It is part of the Jigjiga *One Health Initiative* (JOHI) aiming to create innovative integrated health systems addressing the health and well-being of pastoralist communities.^4,5^

### 2.2. Selection of villages

We conducted the survey in 6 villages of the Shiniile Zone in the SRS of Ethiopia. The villages were randomly selected from 9 eligible villages in the region. To ensure representativeness of the population, the random selection of the 6 villages in the Shiniile Zone was based on the livelihood of the pastoralists (3 villages are pastoralist, and 3 are agropastoralist). We had to exclude 9 other villages because of safety concerns or for reasons of inaccessibility (flooding and lack of transport routes; 3 hours of travel time from the main study camp in Shiniile).

### 2.3. Selection of households in the villages

We applied global positioning system (GPS)-based household localization and recruitment using a tablet-based application for randomly selecting households.^54^ Before the start of data collection, we randomly chose 25 households in each of the 6 villages, using Google Earth. We drew polygons around housing areas within the selected village that were visible through satellite function. Within each polygon, we generated 25 random geocoordinates. Each household received a unique identifier code for the survey. We exported the latitude and longitude coordinates into an Excel sheet. Later, we inserted the GPS locations into the data collection software Open Data Kit (ODK; https://www.opendatakit.org/). To help data collectors locate households, we installed the open-source application Organic Maps on each tablet. We selected this application because it works offline and is compatible with ODK. To locate the prespecified coordinates on foot in the respective village, data collectors followed the route suggested by the application. Once they arrived at the location, they approached the nearest household at the sampling point. If the household refused to participate, they contacted the nearest household in the area.

### 2.4. Sampling of household members

Men and women who were 18 years or older and lived in the sampled household were eligible to participate in the survey. In each household, we aimed to randomly sample 1 female and 1 male resident. If no males or females of the household met the eligibility criteria, we selected 2 males or 2 females, respectively. If there was more than 1 eligible female or male in the household, data collectors randomly chose a participant by drawing a random number attributed to each eligible interviewee. If 1 member of the household refused to participate, they recruited another household member.

### 2.5. Sample size

We aimed for a total sample size of 300 participants, 1 male and 1 female in each household. For the sample size determination, we assumed an average chronic pain prevalence of 20% according to worldwide estimates in general population samples.^53^ This global estimate was selected based on considerations of heterogeneity in chronic pain definitions and prevalence studies.^25,30^ We also considered the fact that data on chronic pain prevalence among pastoralist populations are missing. For our primary objective, we calculated that a sample size of 300 persons would be sufficient to determine the prevalence with a precision—defined as one half-length of the 95% confidence interval (CI)—of 5.2 percentage points. The same sample size would allow us to detect a true difference of the prevalence of 16% points in males and females with 80% power at the 5% significance level, thereby assuming a balanced gender distribution.

### 2.6. Face-to-face interview

The senior of the 2 interviewees per household responded to the household questionnaire. Each interviewee separately answered the questions of the individual part of the questionnaire.

### 2.7. Questionnaire development

Preceding qualitative research (including 2 focus group discussions) with Somali pastoralists in the region and with their treating health professionals helped us to develop the questionnaire.^4,5^ In an additional literature review, we identified studies with comparable populations and research questions providing guidance for potential chronic pain risk factors^59^ and for instruments measuring pain severity and burden, such as the Faces Pain Scale–Revised (FPS-R)^11,21,24^ or the Brief Pain Inventory.^1,2^

The translation and cultural adaptation of the questionnaire (originally designed in English) followed recommendations by Beaton et al.^6,7^ Two Somali mother-tongue speakers independently translated the questions from English into Somali. Two additional translators fluent in both English and Somali performed the back-translation. They discussed translation discrepancies until they reached a consensus. Ten Somali pastoralists pilot-tested the initial survey questions, and 5 Somali pastoralists piloted the final version of the survey. We incorporated minor adjustments to the questionnaire based on the feedback during pilot testing.

Using the data collection software ODK, we designed the survey data. Data collectors downloaded the questionnaire on Android tablets or on their mobile phones for backup by means of the mobile application ODK collect.^37^

### 2.8. Questionnaire domains

The household questionnaire addressed general household information. The individual part of the questionnaire focused on sociodemographic information, followed by illness-related information, affectedness by FGC, and pain-related questions (see Table 1; see questionnaire in Appendix A, http://links.lww.com/PAIN/C63). To consider regular physical work activities for each person with and without chronic pain, we inquired what activities the person has performed on a regular basis in the past 12 months, thereby also referring to physical work activities that might be more common in the rainy or dry season. We then grouped these activities together to differentiate between potentially strenuous activities inside and those outside the home.

**Table 1:** Household and individual questionnaire domains.

Household questionnaire	Number of household members Livelihood (pastoralist; agropastoralist) Livestock (types of animals) Religion (Muslim; other) Ethnicity (Somali; other)
Individual questionnaire	
Sociodemographic information	Gender (male; female) Age (number of years) Education (no formal education; primary; higher) Marital status (single; married) Number of children Regular activities in the past 12 mo* (yes; no)
Illness-related information	Present illness or health problem other than chronic pain† Type of health problem (gastrointestinal problems; respiratory problems; weakness; fever; acute headache; and other) Female genital cutting (FGC) (yes; no)
Pain-related information	Presence of chronic pain (yes; no) Pain body sites (more than one possible) Knee pain (eg, when you walk long distances or fetch water) Lower-back pain (eg, when you are carrying something or when you get up in the morning) Headache (eg, pain on one side of the head, maybe causing concentration problems, vision problems and/or nausea/vomiting) Abdominal pain (eg, you might also lose appetite, feel nauseous and/or feel bloated, maybe feeling worse after eating) Female pain problems (eg, cramping during menstruation or pain related to FGC) Other chronic pain (eg, neck, shoulder, and hips) Pain frequency Persistent (pain is always there) Intermittent/pain attacks (pain comes and goes) Severity‡§ (scale 0-10) Interference‡‖ (scale 0-10)
Biomedical treatment itinerary	Visit to biomedical health facilities
Traditional treatment itinerary	Visit to traditional healers
Social treatment itinerary	Support from family or community
Biomedical pain management	Medication to treat pain
Cultural including religious pain practices	Using holy water Praying Burning one's own skin Slaughtering an animal
Causes of pain	Underlying illness Strenuous work Old age Religious interpretation

* The question was formulated as follows: “What activities did you do regularly in the past 12 months? Also think of things you do more often when it is cold or warm or during the rainy season and the dry season.”

† The question was formulated as follows: “Are you currently suffering from a health problem or from an illness?” (other than chronic pain).

‡ If more than 1 pain body site was reported, the item refers only to the pain reported to be most troublesome.

§ Faces Scale 0 to 10 measuring pain at the time of the interview, average pain, worst pain, and least pain in the past 24 hours. Pain severity score based on the mean value of the 4 severity items.

‖ Verbal scale 0 to 10 interference with activity, with enjoyment in the past 24 h.

To distinguish chronic pain from other illnesses, we described pain to participants as something that can hurt a person but is different from, eg, fever or diarrhea. This distinction was necessary based on the findings from the preceding qualitative interviews and focus group discussions. First and foremost, the Somali word for pain “xanuun” can mean both (chronic) pain and (acute) illness.^4,5,9^ Hence, the participants were informed that the survey focuses on pain that can be described as a very uncomfortable feeling and has been present for more than 3 months. It can impact their daily life, thereby making it more difficult to perform household work or care for their animals.

The questionnaire went on to distinguish what type of chronic pain may be present, eg, knee pain or lower-back pain with further descriptions and examples and how frequent the pain was (persistent or intermittent) (Table 1). When a person reported at least 1 type of chronic pain, more detailed questions were asked (persons with more than 1 pain location identified the pain influencing their daily life the most; subsequent pain-related questions refer only to this pain): (1) Pain severity was verbally assessed using a Faces Scale from 0 to 10. The participants were asked to describe their chronic pain at the time of the interview, their average pain, worst pain, and least pain in the past 24 hours. The pain severity score was based on the mean value of these 4 items. (2) Pain interference with general activity and enjoyment in the past 24 hours was verbally rated on a scale from 0 to 10. (3) Treatment itineraries (biomedical, traditional, and social); personal pain management practices; and beliefs about causes of chronic pain were assessed. The specific questions for participants with chronic pain can be found in Appendix A, http://links.lww.com/PAIN/C63, questions Q14 and onwards.

### 2.9. Data collection

The data collection took place from December 2022 to January 2023. Four trained fieldworkers with a background in nursing and fluent in Somali administered the survey in the Somali language. The 2 male data collectors conducted the survey with the male interviewees, and the 2 female data collectors spoke with the women. We chose this procedure based on findings from preceding qualitative research in the region. Thereby, we wanted to ensure that men and women felt free to respond.^4,5^ We were also careful to speak to women in private, out of earshot of men, whenever possible—in line with recommendations of other research with pastoralists.^15^

A pair of 2 data collectors visited 12 to 13 households per community, amounting to 150 households in total. A village guide accompanied each pair of data collectors. On entering a home together with the guide, the team informed about the survey and its purpose. They obtained written informed consent before commencing with the interview.

The 4 data collectors were trained to upload completed questionnaires to a secure server each evening after data collection. The research team leader had back-end access. To improve data quality, the research team leader provided continuous feedback on the data collected.

### 2.10. Statistical analyses

After completion of the data collection, we cleaned and analyzed all data, using the statistics software Stata. For plotting graphs, we used the software R.

We present the distribution of participant characteristics overall as well as gender-specific as means and percentages, respectively (Table 2 and associated text). To present the overall as well as age- and gender-specific prevalence of chronic pain, we used a 95% confidence interval adjusted for clustering on household level (Table 3 and associated text). The same principles were applied to pain prevalence analyses among women with and without FGC (Appendix C, http://links.lww.com/PAIN/C63).

**Table 2:** Characteristics of the study population, overall and stratified by gender.

Count	Women	Men	Total
	N (%)	N (%)	N (%)
	199 (66.6)	100 (33.4)	299 (100.0)
Age groups			
18-34	104 (52.3)	34 (34.0)	138 (46.2)
35-54	74 (37.2)	45 (45.0)	119 (39.8)
55 or older	21 (10.6)	21 (21.0)	42 (14.1)
Religion			
Muslim	199 (100.0)	100 (100.0)	299 (100.0)
Ethnicity			
Somali	199 (100.0)	100 (100.0)	299 (100.0)
Education			
No formal education	156 (78.4)	63 (63.0)	219 (73.2)
Primary	20 (10.1)	16 (16.0)	36 (12.0)
Higher	23 (11.6)	21 (21.0)	44 (14.7)
Marital status			
Single	57 (28.6)	19 (19.0)	76 (25.4)
Married	142 (71.4)	81 (81.0)	223 (74.6)
Number of children			
0	37 (18.6)	17 (17.0)	54 (18.1)
1 to 3	66 (33.2)	40 (40.0)	106 (35.5)
4 to 6	72 (36.2)	31 (31.0)	103 (34.5)
7 or more	24 (12.1)	12 (12.0)	36 (12.0)
Livelihood			
Agropastoralist	85 (42.7)	39 (39.0)	124 (41.5)
Pastoralist	114 (57.3)	61 (61.0)	175 (58.5)
Regular activities inside the home in the past 12 mo*			
Yes	103 (51.8)	0	103 (34.5)
No	96 (48.2)	100 (100.0)	196 (65.6)
Regular activities outside the home in the past 12 mo†			
Yes	67 (33.7)	51 (51.0)	118 (39.5)
No	132 (66.3)	49 (49.0)	181 (60.5)
Other regular activities in the past 12 mo‡			
Yes	34 (17.1)	38 (38.0)	72 (24.1)
No	165 (82.9)	62 (62.0)	227 (75.9)
Presence of illness or health problem			
Yes	81 (40.7)	38 (38.0)	119 (39.8)
No	118 (59.3)	62 (62.0)	180 (60.2)
Common illnesses or health problems§			
Weakness	20 (24.7)	2 (5.3)	22 (18.5)
Fever	5 (6.2)	10 (26.3)	15 (12.6)
Gastrointestinal problem	7 (8.6)	7 (18.4)	14 (11.8)
Acute headache	8 (9.9)	4 (10.5)	12 (10.1)
Female genital cutting (FGC)			
Yes	122 (61.3)	n.r.	n.r.
No	77 (38.7)		

Percentages may not add to 100% because of rounding.

“n.r.” indicates that the information is not relevant as only women responded to this question.

* Caring for children, cooking, or cleaning.

† Herding or loading animals, carrying water or wood for long distances, and other agricultural work.

‡ Other activities described by the participants (eg, going shopping) and not directly related to agricultural activities or work with livestock.

§ The percentages shown in this section are proportions of those reporting an illness. The numbers do not add up to the total number reporting an illness or health problem because only the most common reported problems are listed.

**Table 3:** Chronic pain prevalence and related aspects overall and by gender.

	Women	Men	Total
Chronic pain prevalence*			
N	69	17	86
%, 95% CI	34.7 (28.4-41.5)	17.0 (10.8-25.7)	27.6 (21.6-34.6)
Locations (body sites)† (percentages shown in this section are proportions of those with chronic pain)			
Knee pain			
N	26	6	32
%	37.7	35.3	37.2
Lower-back pain			
N	26	3	29
%	37.7	17.6	33.7
Headache			
N	14	6	20
%	20.3	35.3	23.3
Female pain‡			
N	6	n.r.	n.r.
%	8.7	n.r.	n.r.
Abdominal/stomach pain			
N	3	3	6
%	4.3	17.6	7.0
Other pain			
N	7	1	8
%	10.1	5.9	9.3
Multiple types of pain			
N	13	2	15
%	18.8	11.8	17.4
Characteristics of chronic pain (percentages shown in this section are proportions of those with the pain at the specific site)			
Knee pain characteristics			
Intermittent			
N	17	3	20
%	65.4	50.0	62.5
Persistent			
N	9	3	12
%	34.6	50.0	37.5
Lower-back pain characteristics			
Intermittent			
N	18	1	19
%	69.2	33.3	65.5
Persistent			
N	8	2	10
%	30.8	66.7	34.5
Headache characteristics			
Intermittent			
N	10	5	15
%	71.4	83.3	75.0
Persistent			
N	4	1	5
%	28.6	16.7	25.0
Female pain characteristics			
Intermittent			
N	5	n.r.	n.r.
%	83.3	n.r.	n.r.
Persistent			
N	1	n.r.	n.r.
%	16.7	n.r.	n.r.
Abdominal/stomach pain characteristics			
Intermittent			
N	2	2	4
%	66.7	66.7	66.7
Persistent			
N	1	1	2
%	33.3	33.3	33.3
Other pain characteristics			
Intermittent			
N	6	0	6
%	85.7	0	75.0
Persistent			
N	1	1	2
%	14.3	100.0	25.0

* Adjusted for the random effect on household level.

† Having more than 1 type of pain was possible; accordingly, the numbers summed over the different sites add up to a higher number than the total number of participants reporting chronic pain.

‡ Pain related to FGC, severe menstrual pain problems.

“n.r.” indicates that the information is not relevant as only women responded to this question.

We chose a random-effect (random effect on household level) multiple logistic regression model to investigate sociodemographic and health factors that are independently associated with chronic pain as outcome variable (Table 4 and associated text). We used the household rather than the village as random effect because an initial analysis of the variance components indicated that the clustering on village level was close to 0. The variables of the multiple logistic regression model are presented in Table 5. All multivariable models were prespecified based on published literature^18,25,30,45,59^ and preceding qualitative research findings.^4,5^

**Table 4:** Independent associations of sociodemographic and health characteristics with self-reported chronic pain.

Chronic pain	Variables	Adjusted odds ratio (95% CI)	*P*
Gender	Female	7.9 (2.2-27.8)	0.001
Age groups	18-34 (ref.) 35-54 55 or older	2.6 (0.9-7.0) 25.2 (4.8-133.2)	0.07 <0.0001
Education groups	No formal education (ref.) Primary Higher	1.9 (0.5-7.1) 1.2 (0.3-4.9)	0.35 0.81
Marital status	Single (ref.) Married	0.5 (0.1-1.9)	0.32
Number of own children	No children (ref.) 1 to 3 4 to 6 7 or more	1.8 (0.3-9.6) 2.1 (0.4-11.6) 1.2 (0.2-8.3)	0.50 0.39 0.87
Livelihood	Agropastoralists (ref.) Pastoralist	1.4 (0.6-3.2)	0.43
Work inside the home	Yes	1.2 (0.4-3.4)	0.78
Work outside the home	Yes	0.7 (0.3-1.7)	0.42
Illness (excluding chronic pain)	Yes	27.0 (8.0-91.0)	<0.0001

**Table 5:** Variables of the multiple logistic regression model.

Outcome variable	
Chronic pain	No [reference] Yes
Predictor variables	
Gender	Male [reference] Female
Age groups	18-34 [reference] 35-54 ≥55
Livelihood	Agropastoralist [reference] Pastoralist
Education groups	No formal education [reference] Primary Higher
Marital status	Single [reference] Married
Number of children	No children [reference] 1-3 4-6 7 or more
Regular activities inside the home in the past 12 mo (including caring for children, cooking, or cleaning)	No [reference] Yes
Regular activities outside the home in the past 12 mo (including herding or loading animals, carrying water or wood for long distances, and other agricultural work)	No [reference] Yes
Presence of another illness or health problem	No [reference] Yes
Female genital cutting (FGC)*	No [reference] Yes

* Appendix D, http://links.lww.com/PAIN/C63.

Associations are reported as adjusted odds ratios and 95% confidence intervals. All analyses described above were additionally run separately in the sample of women, stratified by the presence or absence of FGC or replacing “gender” by “FGC” as predictor variable in the regression model (Appendix D, http://links.lww.com/PAIN/C63). We set the statistical significance level for the *P*-value at *P* < 0.05.

### 2.11. Ethical considerations

The University of Jigjiga Ethical Review Board in the SRS approved this study (Ref. No: RERC/020/2012E.C). The Ethics Committee of Northwest and Central Switzerland (EKNZ) confirmed the fulfilment of ethical and scientific standards (Ref. No: 2020-00338).

## 3. Results

### 3.1. Characteristics of the study population

For this survey, we recruited 150 of 165 approached households and 299 participants. On average, 6 persons lived in every household. The number of persons per household ranged from 2 to 14 (SD: 2.2). The majority of participants owned livestock (81.9%, n = 245), such as goats or sheep. Table 2 shows the distribution of relevant sociodemographic variables overall and stratified by gender.

Most participants were female (66.6%, n = 199) and pastoralists (58.5%, n = 175). The mean age was 38 years, ranging from 18 to 90 years (SD: 15.5). All participants were from the Somali ethnic group and belonged to the Muslim religion. Most participants had no formal education (73.2%, n = 219). Exclusively women reported working inside the home, whereas men and women declared working outside the home. Approximately 51.8% (n = 103) of women were engaged in regular activities inside the home in the past 12 months (caring for children, cooking, or cleaning). Approximately 33.7% (n = 67) of women reported working outside the home in the past 12 months, such as herding animals or agricultural work. Approximately 39.8% (n = 119) of participants reported that they were suffering from a health problem other than chronic pain, such as general weakness, fever, or gastrointestinal complaints. The majority of women (61.3%, n = 122) reported having undergone FGC.

When comparing women who had undergone FGC with those who had not, more women with FGC had no formal education (62.8%, n = 98) compared with women without FGC (37.2%, n = 58) (Appendix B, Table B, http://links.lww.com/PAIN/C63).

### 3.2. Prevalence of chronic pain

The random-effect model estimated an overall chronic pain prevalence of 27.6% (95% CI: 21.6-34.6). However, it varied significantly between ages and genders. It increased from 5.4% (95% CI: 0.8-30.1) for those aged between 18 and 34 years (n = 138) to 27.1% (95% CI: 15.1-43.7) for participants aged between 35 and 54 years (n= 119) and to 69.1% (95% CI: 53.7-81.1) for those aged 55 years and older (n = 42). Women were nearly twice as likely to report chronic pain (34.7% [95% CI: 28.4-41.5]) compared with men (17.0% [95% CI: 10.8-25.7]) (Table 3). Women who had not experienced FGC were more likely to report chronic pain (46.8% [95% CI: 35.9-57.9]) compared with women who had undergone FGC (27.1% [95% CI: 19.9-35.6]) (see Appendix C, Table C, http://links.lww.com/PAIN/C63), although the result was not statistically significant.

**Figure 1.**
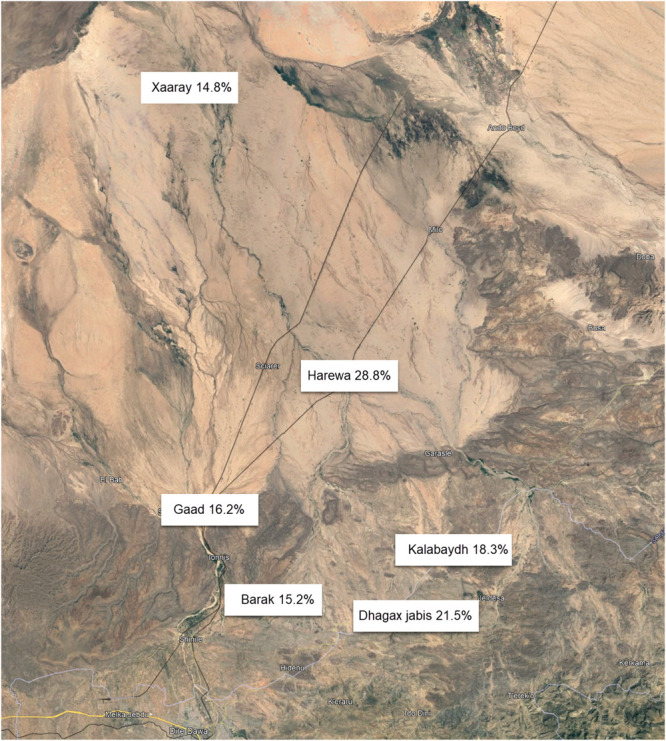
Age- and gender-adjusted point estimate of chronic pain prevalence per village.

When comparing age- and gender-adjusted prevalence between the 6 different villages, Harewa (28.8% [95% CI: 22.3-35.2]) and Dhagax Jabis (21.5% [95% CI: 16.2-26.9]) had the highest prevalence of chronic pain compared with Xaaray village (14.8% [95% CI: 8.9%-20.7%]) with the lowest prevalence (Fig. 1).

### 3.3. Characteristics of chronic pain: location, severity, frequency, and impact

The pain body sites and other pain characteristics among women and men reporting chronic pain are also presented in Table 3. Knee pain (37.2%), lower-back pain (33.7%), and headache (23.3%) were the body sites most commonly affected across both genders among those reporting chronic pain. This amounted to a pain prevalence for each pain location in the total study sample of 10.7% for knee pain, 9.7% for lower-back pain, and 6.7% for headache. Approximately 5.0% of the total study sample reported pain in more than 1 location. Among those with chronic pain, lower-back pain was much more prevalent among women (37.7%) compared with men (17.6%). As opposed to lower-back pain, headache was more common among men (35.3%) compared with women (20.3%) among those with chronic pain.

Regarding the body sites most frequently affected by pain, we found that most women reported intermittent knee or lower-back pain, whereas one-third of women said that their chronic pain (eg, knee, lower back, and head) was persistent.

Most women with chronic pain reported mild (2-4) average pain severity over the past 24 hours (61.8%, n = 42) followed by moderate (6) pain (29.4%, n = 20). Regarding FGC, 42.4% (n = 14) of women with chronic pain who had undergone FGC reported moderate pain (6) compared with 17.1% (n = 6) of women who had not undergone FGC. Men with chronic pain most commonly reported a moderate average pain severity (52.9%, n = 9). In the case of more than one type of pain, these severities refer to the pain site that was considered most troublesome by the participant.

The majority of men (58.8%, n = 10) with chronic pain stated that their painful condition impacted their overall activity on a level of 4 to 6 (on a scale of 0 = “no interference” to 10 = “most interference”). In comparison, 50.7% of women (n = 35) with chronic pain reported the same level of activity inference. Approximately 21.7% (n = 15) of women with chronic pain reported that pain severely affected their activity (scale 8-10), compared with 35.3% (n = 6) of men. Again, in the case of multiple affected sites, the results relate to the pain site considered most troublesome.

**Figure 2.**
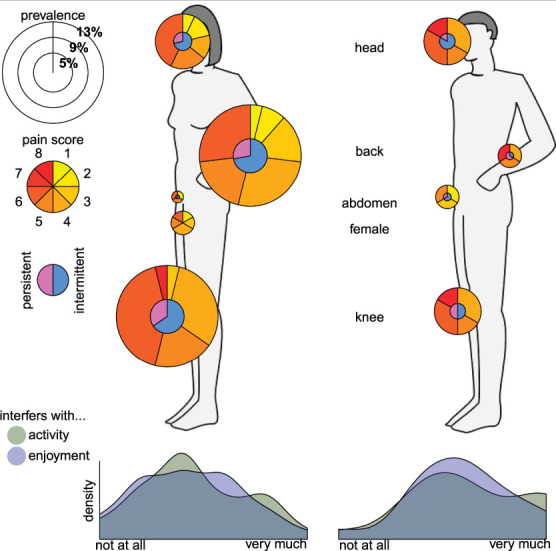
Chronic pain prevalence and affected body site as well as severity (pain severity score), frequency (intermittent vs persistent), and interference in women and men with chronic pain.

Figure 2 visualizes how the dimensions of chronic pain burden (prevalence, location/body site, severity, frequency [intermittent vs persistent], and impact/interference) are distributed, separate for women and men.

### 3.4. Sociodemographic and health factors associated with chronic pain

The results of multiple logistic regression analysis with mutually adjusted associations of sociodemographic and health factors with the presence of chronic pain are summarized in Table 4. The reporting of chronic pain was associated with female gender (OR: 7.9 [95% CI: 2.2-27.8] compared with men), being aged 55 years or older (OR: 25.2 [95% CI: 4.8-133.2]) compared with 18 to 34 years) and having another illness or health problem (OR: 27.0 [95% CI: 8.0-91.0 compared with no other illness]). The prevalence of chronic pain was not statistically significantly associated with education, marital status, number of children, livelihood, or potentially strenuous repetitive work activities inside or outside the home. Furthermore, FGC was not statistically significantly associated with chronic pain (Appendix D, Table D, http://links.lww.com/PAIN/C63).

### 3.5. Treatment itineraries and therapeutic practices

**Figure 3.**
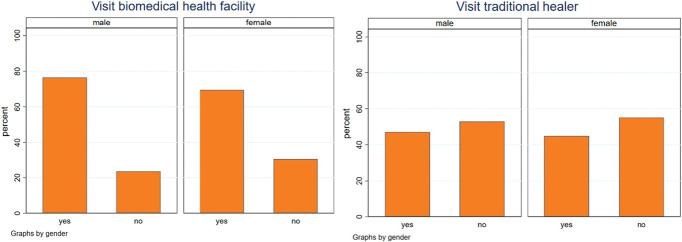
Treatment practices for chronic pain by gender.

Regarding the biomedical treatment itinerary, we found that the majority of participants with chronic pain stated having visited a biomedical health facility (including pharmacy) to seek treatment for their pain (70.9%, n = 61). A slightly higher percentage of men attended a biomedical health facility compared with women (Fig. 3). In addition, nearly half of the participants with chronic pain had also visited a traditional healer in the past to treat their pain (45.3%, n = 39). When comparing women with and without FGC and suffering from chronic pain, 87.9% (n = 29) of women with chronic pain who had undergone FGC visited a biomedical health facility compared with 52.8% (n = 19) who had not undergone FGC (Appendix E, Figure E1, http://links.lww.com/PAIN/C63).

The most common reasons for not visiting a health facility were that the chronic pain was not severe enough, the facility was difficult to reach, or the costs of treatment were too high.

**Figure 4.**
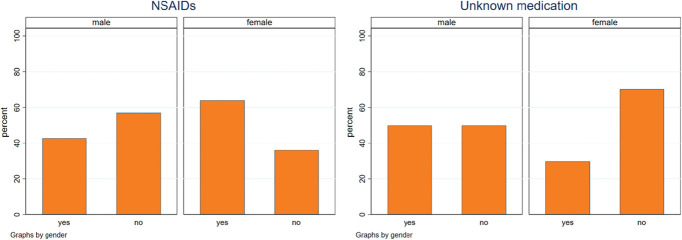
Biomedical treatment (medication) by gender.

The majority of participants with chronic pain reported having taken medication to treat their pain (90.7%, n = 78), most commonly nonsteroidal anti-inflammatory drugs (NSAIDs) (60.3% of those with chronic pain taking medication, n = 47). One-third of participants with chronic pain who had taken medication did not know the name of the drug (33.3%, n = 26) (Fig. 4). For women with FGC taking medication for their chronic pain, 46.9% (n = 15) reported taking NSAIDs, compared with 81.3% (n = 26) of women who had not undergone FGC (Appendix E, Figure E2, http://links.lww.com/PAIN/C63).

**Figure 5.**
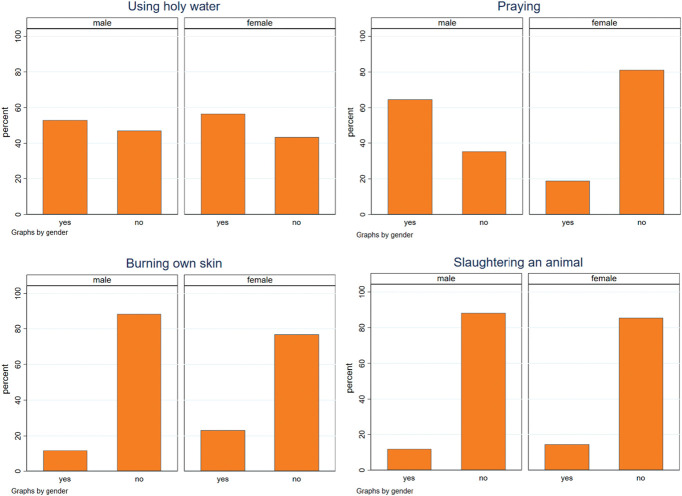
Cultural including religious practices to deal with chronic pain by gender.

To deal with their chronic pain, cultural including religious practices were also common among participants (Fig. 5). More than half of participants with chronic pain reported using holy water for pain relief (55.8%, n = 48), followed by praying (27.9%, n = 24). One-fifth of participants with chronic pain also reported to burn their own skin (20.9%, n = 18) or to slaughter an animal (14.0%, n = 12) to cope with pain. However, participants reported that none of these practices had completely relieved their pain in the past month.

Women with FGC more often reported using holy water and praying to deal with pain compared with women who had not undergone FGC (Appendix E, Figure E3, http://links.lww.com/PAIN/C63).

Within the family, women were most often supported by their daughters regarding pain problems (40.6%, n = 28), followed by their husbands (24.6%, n = 17), whereas men were most commonly supported by their wives (58.8%, n = 10).

Regarding the causes of pain, the most common answer among those with chronic pain was that having this pain was a destiny from Allah (39.5%, n = 34), followed by work activities perceived to be strenuous (29.1%, n = 25).

## 4. Discussion

We investigated chronic pain in a random sample of adult pastoralists in the SRS. In the following discussion of our main results, we address (1) the overall prevalence in context, (2) the location and burden of pain, (3) gender differences, (4) treatment itineraries, and (5) implications concerning the *One Health Initiative.*

### 4.1. Overall prevalence in context

In this study, we identified a chronic pain prevalence of 27.6% among adult pastoralists. Although data on pain prevalence are very heterogeneous and definitions vary,^25,39^ certain cautious comparisons are possible. Kamerman et al.^30^ found a 18.3% overall prevalence of chronic pain in their recent nationally representative study in the South African population. Furthermore, Jackson et al.^25^ reported in their systematic review and meta-analysis that the average prevalence of unspecified chronic pain in low- and middle-income countries was 34% in the general adult populations and 62% in the older general populations.

### 4.2. Location and burden of pain

The most common body sites affected by chronic pain were knees (37.2%) and lower back (33.7%). This is comparable with the South African study reporting limb pain to be the most common pain location (43.6%), followed by back pain (30.5%).^30^

Although most women in our study affirmed having undergone FGC, very few women reported any gynecological pain. This is not to say that severe chronic pain as a result of FGC was not present in women. On the contrary, Perović et al.^45^ found that in qualitative interviews, Somali women with FGC described debilitating pain in daily life. However, the authors argued that verbal assessment tools, such as the McGill Pain Questionnaire, did not capture this level of severity. In our study, we also identified that pain severity on all body sites was rarely considered to be above moderate intensity, despite the fact that none of the available pain management practices resulted in complete pain relief. One explanation is the stoic attitude towards pain that is characteristic for Somali culture^4,49^ and common among pastoralist populations.^48,57^ In addition, we found that most respondents reported that their chronic pain moderately interfered with their activity or overall enjoyment. Moreover, men reported a higher negative impact compared with women. Our preceding qualitative research indicated a very high self-perceived burden of chronic pain in daily life among both genders. Women mentioned that they were no longer able to care for their children. Men reported difficulties in looking after their livestock and financially supporting their families.^4^ This highlights the importance of culturally sensitive pain assessment tools capturing the multidimensionality of chronic pain experiences in this particular cultural and ecological context.

### 4.3. Gender differences

Our results reveal that women were faced with a much higher prevalence of chronic pain than men. Female vulnerability towards bad health outcomes in pastoralist communities has been highlighted in the literature^3^ in the context of gendered health inequalities in Ethiopia. One aspect that is well worth emphasizing is the high prevalence of FGC in this study. This result corresponds with other study findings in the SRS,^17^ although chronic pain was not significantly associated with this practice. Furthermore, female participants of our survey were not only engaged in (potentially strenuous) household activities as opposed to men. They also cared for livestock and undertook agricultural activities. This possibly contributes to their elevated risk of chronic pain. However, we found no statistically significant effect of certain pastoralist or agropastoralist activities on chronic pain prevalence. In the South African context, Geere et al.^18^ argue that physical strain (eg, carrying water) can potentially lead to musculoskeletal disorders and disability. These household activities are mainly performed by women. In addition, previous studies have determined a positive association between the number of children and younger age at first childbirth with the prevalence of chronic lower-back pain.^20,60^

### 4.4. Treatment itineraries

In our study, participants with chronic pain had a surprisingly high access to biomedical care. Most participants with chronic pain stated that they had already sought care in a health facility. However, the accessibility to health facilities and medications differed significantly between villages. One explanation is linked to infrastructure: Some villages were closer to major roads or railway stations than others.

Most participants with chronic pain reported using NSAIDs, women more than twice as often. However, our data do not determine whether NSAIDs were obtained over the counter (OTC) or on prescription. A recent scoping review^31^ found that high OTC use of analgesics is a very common practice in sub-Saharan Africa. According to the review, the main reasons for using OTC drugs were difficulties in accessing professional health services or perceiving the health problem to be minor. Moreover, self-medicating was also more common among women and persons with low education levels. In our preceding qualitative study, we found that pastoralists indeed had access to (primary) biomedical health services. However, the quality of services was often poor, especially in rural areas with very limited resources. Moreover, self-medicating sometimes resulted in gastrointestinal problems.^4^

The findings of our current survey indicate that nearly half of the participants with chronic pain had visited a traditional healer to treat their pain. This result is in line with a study in Southern Ethiopia, where 40.5% of pastoralists attended a traditional healer in response to illness.^32^ Furthermore, spiritual practices were also common. Previous studies among Somali pastoralists have considered such practices in light of the belief in the divine source of all health problems.^4,10^ In line with previous research in the SRS, our findings highlight the existence and importance of medical plurality among pastoralists, with biomedical, traditional (eg, burning the painful area), and spiritual health practices (eg, slaughtering of an animal) existing in parallel.^4,5,10^

### 4.5. The *One Health* implications of pain

In the context of the *One Health Initiative*, our results underline the importance of linking human and animal health in pastoralist contexts. The pastoralists' main source of income is animal husbandry. This requires a high level of physical activity for herding, milking, watering, and caring for animals. Chronic pain directly affects all physical activities associated with livestock husbandry. It directly leads to reduced income and food security. Zoonotic diseases such as brucellosis transmitted from livestock can also lead to debilitating chronic pain, if not recognized timely.^47^ In turn, lack of attention to animals because of pastoralists' chronic pain causes poor animal health and mortality. Hence, there are important secondary effects of chronic pain on animal health as well as on human production and food security.

## 5. Limitations and strengths

We considered chronic pain to be present if an interviewee confirmed it verbally. We cannot exclude any social desirability bias linked to certain reports made by participants. According to the literature, the reliability and validity of self-reported pain in surveys are acceptable.^28^ To limit potential bias, we informed the participants that their answers would not have any negative or positive consequences. There was no compensation for participating in the survey (eg, by receiving medication). Almost twice as many women as men participated and gender balance was not achieved. Limitations in statistical power are still present and evidenced in wide confidence intervals, particularly in the multiple logistic regression analysis. The independent associations of predictors with chronic pain are therefore not conclusive. Moreover, the study is only focused on 1 zone in the SRS. Therefore, the transferability of the results to other settings is not straightforward. Despite these limitations, the survey may guide the implementation of equivalent investigations in other settings.

To the best of our knowledge, this is the first study investigating chronic pain prevalence and burden among Somali pastoralists in a community setting. Although the sample size was small, the rigorous sampling approach offers initial insights into the chronic pain burden of this marginalized community. The broad definition of chronic pain allows comparisons with other studies. Furthermore, the information on treatment itineraries can foster the development of future pharmacological and particularly nonpharmacological pain-relieving interventions. The development of a specific questionnaire in a mixed-methods approach and its rigorous pilot testing ensured appropriate cultural adaptations. The earlier qualitative interviews allowed for the reflection on the meaning of the interviewees' answers in the subsequent quantitative survey. Finally, we conducted this study in a setting that is not easily accessible. In this region, pastoralists have faced numerous droughts and disease outbreaks over the past decades, as well as interclan and interstate conflicts.^5,10^

## 6. Conclusion

This study provides novel insights into different domains of chronic pain burden among Somali pastoralists. Based on the results, older persons and women are especially at risk of developing chronic pain. Therefore, they should be considered a priority group for health interventions. Our study also found that chronic pain had a distinct psychosocial impact on participants' lives. Given the tight links of mental health with chronic pain, future health initiatives and treatments should target both aspects. Health professionals and other stakeholders should be aware that chronic pain not only affects the well-being of pastoralists themselves. It also compromises the health of their animals and therefore their livelihoods and those of their communities.

## Conflict of interest statement

The authors have no conflicts of interest to declare.

## Appendix A. Supplemental digital content

Supplemental digital content associated with this article can be found online at http://links.lww.com/PAIN/C63.
